# Factors influencing digital health literacy in stroke patients based on social cognitive theory: a mixed-methods systematic review

**DOI:** 10.3389/fpubh.2026.1905179

**Published:** 2026-08-17

**Authors:** Xiang Li, Pingping Feng, Min Gao, Yuan Yuan, Yingge Wang

**Affiliations:** 1 School of Nursing, Faculty of Medicine, Yangzhou University, Yangzhou, China; 2 Department of Rehabilitation Medicine, Affiliated Hospital of Yangzhou University, Yangzhou, China

**Keywords:** digital health literacy, influencing factors, mixed-methods, social cognitive theory, stroke, systematic review

## Abstract

**Background:**

Digital health literacy is crucial for stroke patients to effectively access and utilize digital health information and services, thereby facilitating health management and improving health outcomes. However, digital health literacy among stroke patients is often inadequate and influenced by multiple factors. This study aimed to systematically identify the factors associated with digital health literacy in stroke patients, so as to provide evidence for the development of tailored intervention strategies.

**Methods:**

Eight databases (PubMed, Cochrane Library, Web of Science, Embase, SinoMed, China National Knowledge Infrastructure, Wanfang, VIP) were searched from inception to April 2026. The eligible studies were synthesized using the convergent integrated approach recommended by the Joanna Briggs Institute (JBI), and the integrated findings were subjected to thematic analysis guided by the Social Cognitive Theory (SCT).

**Results:**

A total of 16 studies were included (12 quantitative studies and 4 qualitative studies). Guided by the Social Cognitive Theory, the synthesized findings indicated that the factors influencing digital health literacy in stroke patients could be classified into three dimensions: personal, environmental, and behavioral factors. Personal factors encompassed sociodemographic and health-related characteristics, psychological and self-regulation characteristics, and technology-related attitudes and digital health capability. Environmental factors included family and social support as well as the information and service environment. Behavioral factors comprised proactive information behaviors and interactive and communication behaviors.

**Conclusion:**

Digital health literacy in stroke patients is influenced by the combined effects of personal, environmental, and behavioral factors. In clinical practice, particular attention should be given to digitally disadvantaged populations, and targeted interventions tailored to patients’ individual characteristics and digital health needs should be implemented to improve their digital health literacy and capacity to use digital health resources. Further high-quality studies are warranted to inform the development of precise intervention strategies and the optimization of rehabilitation management.

**Systematic review registration:**

https://www.crd.york.ac.uk/PROSPERO/view/CRD420261412446, identifier PROSPERO (CRD420261412446.

## 1 Introduction

Stroke is a cerebrovascular disease caused by the rupture or occlusion of intracranial blood vessels, leading to ischemia and hypoxia in local brain tissue. It is associated with high incidence, disability, mortality, recurrence, and economic burden. Globally, stroke remains the second leading cause of death and the third leading cause of disability-adjusted life years (DALYs) among non-communicable diseases (1). Stroke survivors often suffer from motor, speech, cognitive, and psychological impairments, leading to limitations in activities of daily living and reduced quality of life, and therefore require long-term and ongoing health management and rehabilitation support (2, 3).

With the widespread adoption and application of the Internet, an increasing number of individuals obtain information and use online services through digital platforms. According to the 57th Statistical Report on China’s Internet Development, by December 2025, the number of Internet users in China had reached 1.125 billion, with an Internet penetration rate of 80.1%. Meanwhile, the number of online healthcare users reached 411 million, accounting for 36.5% of the total Internet population (4). In recent years, digital health services, such as telerehabilitation, mobile health, wearable devices, and online follow-up, have evolved rapidly and have played an increasingly important role in stroke prevention, diagnosis, treatment, and rehabilitation (5, 6), emerging as an important approach to improving healthcare accessibility and the continuity of rehabilitation. These digital health interventions not only improve stroke patients’ adherence to rehabilitation exercises, self-management capacity, and activities of daily living, but also enhance their knowledge and satisfaction, while providing new opportunities for post-discharge continuity of care (6–9). However, stroke patients’ ability to fully utilize and benefit from health information and services available through the Internet, mobile applications, and other electronic platforms depends largely on their capacity to effectively access, understand, evaluate, and apply digital health resources. This capacity is reflected in digital health literacy.

Digital health literacy is defined as the capacity to translate health knowledge acquired from digital environments into actions, and its conceptual attributes are four, namely goal-driven regulation, information processing, communication and utilization (10). For the purposes of this review, digital health literacy was understood as a broad construct encompassing patients’ ability to access, understand, appraise, communicate, and use digital health information and services. In this sense, it was treated as broader than eHealth literacy, which has traditionally emphasized the ability to seek, find, understand, and appraise health information from electronic sources, and broader than mobile health literacy, which focuses more specifically on literacy in mobile-device-based health contexts. Evidence suggests that adequate digital health literacy can help patients better manage their own health conditions and improve health-related behaviors (11–13). For stroke patients, identifying the key determinants of digital health literacy is essential for the development of digital intervention strategies in neurorehabilitation. Existing studies have primarily examined the factors influencing digital health literacy from either a quantitative or a qualitative perspective alone, which makes it difficult to fully reflect its complexity. By integrating different types of evidence, systematic reviews of mixed-methods studies may offer a more comprehensive and in-depth understanding (14). Social Cognitive Theory (SCT) emphasizes the dynamic interplay among personal, environmental, and behavioral factors. Accordingly (15), this study adopted SCT as its theoretical framework to analyze and synthesize the factors influencing digital health literacy among stroke patients, thereby providing a theoretical basis for interventions aimed at improving their digital health literacy.

## 2 Methods

### 2.1 Search strategy

Eight databases, including PubMed, the Cochrane Library, Web of Science, Embase, SinoMed, China National Knowledge Infrastructure (CNKI), Wanfang, and VIP Database, were searched from inception to April 2026. A combination of subject headings and free-text terms was used. No language restrictions were applied during the database searches; however, during the study selection process, only studies published in English or Chinese were considered eligible for inclusion. The search strategy for PubMed is provided as an example in Supplementary Table S1. The protocol was registered with PROSPERO (CRD420261412446).

### 2.2 Inclusion and exclusion criteria

This review included quantitative, qualitative, and mixed-methods studies and adopted the PICo framework (Population, phenomenon of interest, and context) to define inclusion criteria, ensuring a comprehensive exploration of the topic. The inclusion criteria were as follows: (1) Population: Adults (≥18 years) diagnosed with stroke. (2) Study design: Quantitative, qualitative, or mixed-methods studies. Quantitative studies included those reporting the level, profile, correlates, predictors, or associated factors of digital health literacy among stroke patients. Qualitative studies included those exploring stroke patients’ experiences or perspectives related to digital health information or digital health service use, such as telerehabilitation, video follow-up, remote consultation, or other forms of virtual care, when these findings were relevant to patients’ use of digital health information and services and provided insight into factors related to digital health literacy in practice. (3) Phenomenon of interest: Factors influencing digital health literacy among individuals with stroke. In this review, digital health literacy refers to patients’ ability to access, understand, appraise, communicate, and apply health information and services in digital environments. Eligible factors included both direct influences on these capabilities, as well as factors related to how they are enacted in practice, such as accessibility, usability, informational support, interaction with healthcare providers, technological barriers, and opportunities to use digital resources. (4) Language: Studies published in English or Chinese. The exclusion criteria were as follows: reviews, editorials, conference abstracts, and studies for which full-text data were not accessible.

### 2.3 Quality appraisal

Study quality was assessed using the Mixed Methods Appraisal Tool (MMAT) (16), which enables the appraisal of qualitative, quantitative and mixed methods studies (17, 18). Two reviewers independently performed the quality assessment, and disagreements were adjudicated by a third reviewer. To maximize the inclusiveness and comprehensiveness of the synthesis, no eligible studies were excluded on the basis of methodological quality, allowing the review to reflect the full range of available evidence.

### 2.4 Study selection and data extraction

After a literature search by two authors, the obtained studies were imported into Rayyan to identify and delete duplicate literature. Two reviewers independently screened the titles and abstracts to identify potentially eligible studies, and then assessed the full texts for final inclusion. If two people disagree, they discuss this with the third researcher to reach a consensus. Data were extracted using the Joanna Briggs Institute (JBI) data extraction form (19). The extracted information included the first author, year of publication, country, study design, study objective, sample information, data collection methods, and the reported qualitative and quantitative findings.

### 2.5 Data synthesis and integration

Data were analyzed and synthesized using the convergent integrated approach recommended by the JBI methodology for mixed-methods systematic reviews (20). Quantitative and qualitative data were first extracted separately. The quantitative findings were then transformed into qualitative themes, categories, or narrative descriptions, as JBI recommends qualitizing quantitative data to minimize error and facilitate coherent integration with qualitative evidence. Specifically, quantitative findings were qualitized by translating them into textual descriptions or declarative statements that captured their meaning in relation to the review question. These qualitized data were then assembled with the qualitative findings and grouped into categories and themes based on similarity in meaning. Guided by SCT framework (15), two reviewers independently conducted thematic analysis using directed content analysis based on the key concepts of the framework. The resulting categories and themes were then organized under the relevant SCT components according to their conceptual fit. Any disagreements were resolved through discussion with a third reviewer until consensus was achieved.

## 3 Results

**Figure 1:**
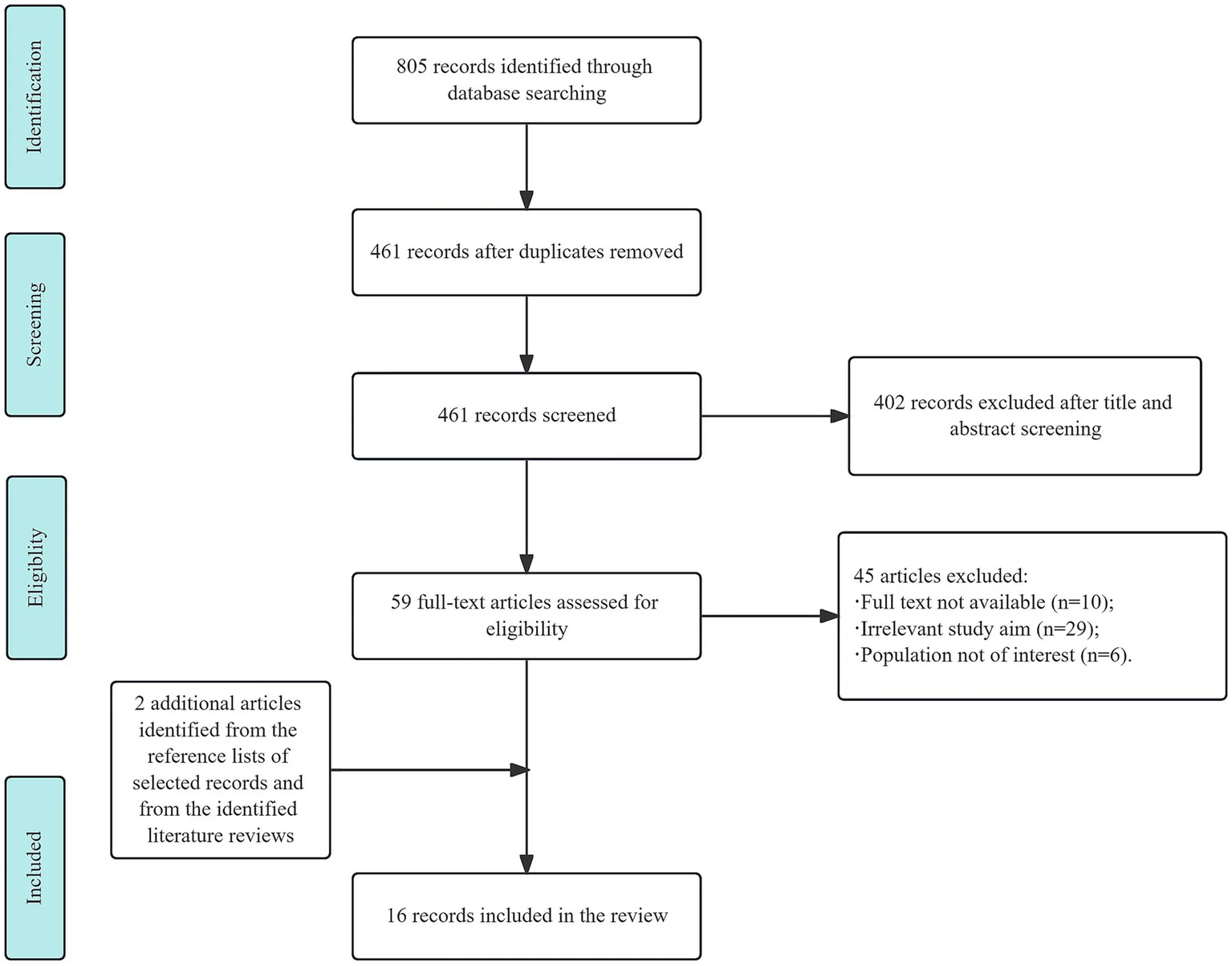
Flowing diagram of included studies selection process.

A total of 805 records were identified through the initial database search. After duplicate removal and title and abstract screening, 59 articles were retrieved for full-text review. In addition, two studies were identified through manual searches of the reference lists. Ultimately, 16 studies were included in the final analysis. Figure 1 shows the process of study selection.

### 3.1 Characteristics of included studies

Table 1 presents the key characteristics of the included studies. This review included 16 studies examining factors influencing digital health literacy among stroke patients, published between 2020 and 2025. The studies were conducted across six countries or regions: mainland China (*n* = 10) (21–30), Hong Kong, China (*n* = 1) (31), South Korea (*n* = 1) (32), Norway (*n* = 1) (33), Canada (*n* = 2) (34, 35), and the United States (*n* = 1) (36). Most were quantitative studies (*n* = 12), while four were qualitative. Sample sizes ranged from 5 to 659 participants, with a total reported sample size of 4,232. The study populations mainly consisted of patients with stroke, with some studies specifically focusing on subgroups such as older adults, young and middle-aged adults, hospitalized patients, and patients with acute ischemic stroke. The included studies primarily examined the current status, potential profiles, and influencing factors of digital health literacy among stroke patients, as well as its relationships with self-efficacy, self-management, social support, and quality of life. A small number of qualitative studies explored patients’ experiences with digital health services, including telerehabilitation, video follow-up, and remote nursing consultation. These studies were relevant to the review because they provided insight into factors related to stroke patients’ use of digital health information and services in practice, particularly acceptability, usability and accessibility, and perceived benefits and barriers.

**Table 1:** The key characteristics of the included studies.

Author (Year)	Region	Study design	Aim	Sample size	Data collection/Tools
Cho et al. (2025) (32)	Korea	quantitative	To identify mobile health literacy and self-care education needs of stroke patients, and to analyze the differences in these needs between groups based on ICT utilization willingness.	100	Demographics, ICT Utilization and Education Needs, eHEALS, Self-Care Education Needs
Linnestad et al. (2025) (33)	Norwegian	quantitative	To identify digital health literacy (DHL) profiles among stroke survivors and examine possible associations with sociodemographic and clinical characteristics.	177	Demographics, eHLQ, Mini-MoCA
Wong et al. (2025) (31)	Hong Kong	qualitative	To explore stroke survivors’ experiences with a nurse-led teleconsultation program, including perceived capabilities, limitations in usability/accessibility/clinical function, and to generate patient-informed recommendations for improvement.	21	Focus groups, thematic analysis based on Braun & Clarke’s framework
Xue et al. (2025) (21)	China	quantitative	To explore latent categories of electronic health (e-health) literacy among stroke patients and analyse its influencing factors.	558	Demographics, eHEALS
Zhang et al. (2025) (22)	China	quantitative	To investigate the status and influencing factors of digital health literacy among stroke patients	332	Demographics, digital health literacy questionnaire for stroke patients (developed by the researcher), HBQ-OAS, SECD6
Duan (2024) (23)	China	quantitative	To investigate the current status and influencing factors of e-health literacy, self-efficacy, and self-management in stroke patients	307	Demographics, eHEALS, SSC, SSMS
Hu (2024) (24)	China	quantitative	To understand the factors influencing e-health literacy in older adults with stroke	106	Demographics, Eheals, HIBS, ISES
Zhang (2024) (25)	China	quantitative	To examine the current status and interrelationships among electronic health literacy, social support, positive psychological capital, and health self-management ability in patients with acute ischemic stroke.	301	Demographics, AHSMSRS, SSRS, eHEALS, PPQ
Liu (2023) (26)	China	quantitative	To investigate the current situation of ehealth literacy of stroke patients in China and analyze its influencing factors	260	Demographics, Barthel Index, eHEALS (developed by the researcher)
Wang (2023) (27)	China	quantitative	To understand the status quo and influencing factors of e-health literacy and quality of life in young and middle-aged stroke patients.	248	Demographics, eHEALS, HBS-SP, SF-36
Yu et al. (2023) (28)	China	quantitative	To investigate the status and influencing factors of e-health literacy in young and middle-aged stroke patients	659	Demographics, eHEALS, Barthel Index, HAMA, HAMD, SSSMQ
Gaboury et al. (2022) (34)	Canada	qualitative	To describe the acceptability of a stroke telerehabilitation platform from the perspective of both patients and therapists.	5	semi-structured interview, hybrid inductive and deductive approach analysis
He (2022) (29)	China	quantitative	To investigate electronic health literacy in Chinese stroke patients, classify patients according to e-health literacy scores, and analyze factors associated with the classification.	564	Demographics, e-HLS, SSRS, GSES
Chan et al. (35) (2021)	Canada	qualitative	The study sought to explore the experiences of participants affected by stroke with home video visit (HVV) for follow-up visits in order to understand the determinants, barriers, and benefits associated with HVVs.	23	semi-structured interview, thematic coding analysis
Zhang et al. (2021) (30)	China	quantitative	To investigate the status and influencing factors of eHealth literacy in hospitalized patients with stroke	558	Demographics, eHEALS
Chen et al. (36) (2020)	USA	qualitative	To investigate patient perceived benefits of and barriers to using the telerehabilitation system at home	13	Semi-structured interviews, thematic analysis based on UTAUT model

eHEALS, e-Health Literacy Scale; SSC, Self-efficacy Scale for Chronic; SSMS, Stroke Self-Management Scale; HIBS, health information seeking behavior; ISES, information self-efficacy scale; SSRS, Social Support Rating Scale; PPQ, positive psychological capital scale; AHSMSRS, The Rating Scale Of Health Self-Management Skill For Adults; HBS-SP, Health Behavior Scale for stroke patients; SF-36, The MOS 36-item short-form health survey; SSSMQ, Southampton Stroke Self -Management Questionnaire; HAMA, Hamilton Anxiety Scale; HAMD, Hamilton Depression Scale; e-HLS, electronic health literacy scale; GSES, general self-efficacy scale; eHLQ, eHealth Literacy Questionnaire; Mini-MoCA, Mini Montreal Cognitive Assessment; SECD6, Self-efficacy for Chronic Disease 6-Item Scale; HBQ-OAS, Health Behavior Questionnaire for Older Adults With Stroke.

### 3.2 Quality assessment

A detailed summary of the quality appraisal for all included studies is shown in Supplementary Table S2. Overall, the methodological quality of the included studies ranged from moderate to high, with most studies showing generally good methodological rigor. Some studies had minor methodological limitations, primarily related to ambiguity in the alignment between data collection, analysis, and interpretation in qualitative research, and insufficient control of confounding factors in quantitative research. No study was judged to be of low methodological quality. Importantly, no major theme was supported solely by studies with relatively lower MMAT ratings. Taken together, these findings suggest that the overall study quality supports a moderate-to-high level of confidence in the review findings, although some caution is warranted when interpreting results from studies with the limitations noted above.

### 3.3 Factors influencing digital health literacy in stroke patients

**Figure 2:**
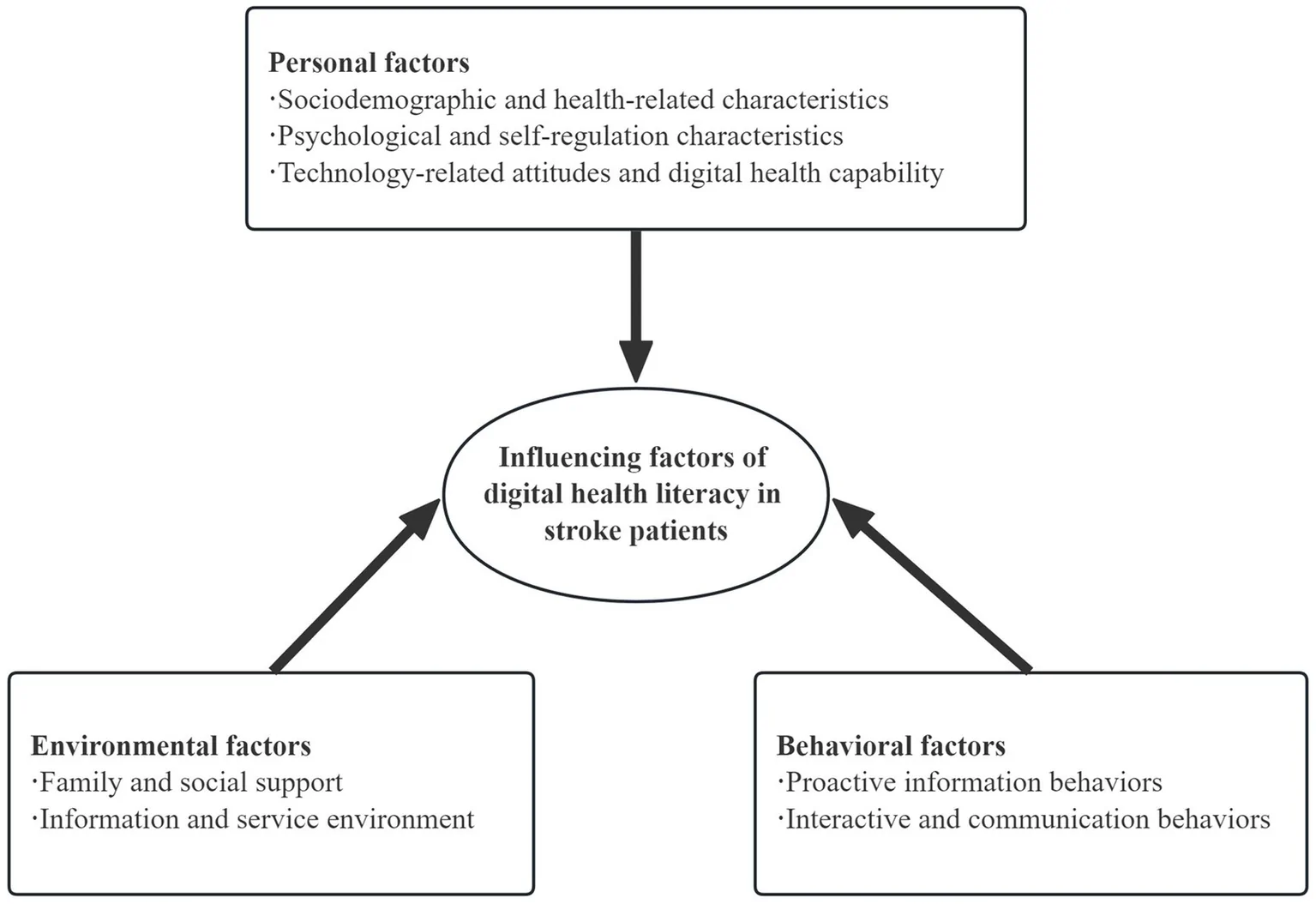
Framework of factors influencing digital health literacy among stroke patients.

Based on the triadic reciprocal model of SCT (15), the findings are presented in terms of personal factors, environmental factors, and behavioral factors. A detailed summary of these factors is presented in Figure 2 and the detailed synthesis of factors derived from the included studies is provided in Supplementary Table S3.

#### 3.3.1 Personal factors

Personal factors encompassed sociodemographic and health-related characteristics, psychological and self-regulation characteristics, and technology-related attitudes and digital health capability.

##### 3.3.1.1 Sociodemographic and health-related characteristics

Regarding sociodemographic and health-related characteristics, eight studies identified age as a factor influencing digital health literacy among stroke patients (22, 23, 25, 27–30, 33), with older patients tending to have lower levels of digital health literacy. Eleven studies supported educational level as an important determinant (21–30, 33), indicating that patients with higher levels of education had better digital health literacy. Eight studies found that better financial status was associated with higher digital health literacy (22, 23, 25–30). Five studies suggested that stroke patients living in urban areas may have higher levels of digital health literacy than those living in rural areas, possibly because of greater access to resources (22–25, 29). Four studies reported that employed patients had higher levels of digital health literacy than those who were retired or unemployed (22, 25, 28, 33). In addition, occupation type also influenced digital health literacy to some extent (24, 27, 30), with cadres and office employees showing higher levels than farmers. One study suggested that unmarried stroke patients had higher levels of digital health literacy (27), whereas married and widowed patients had lower levels. However, another study found that patients with a spouse had higher digital health literacy (29). Other factors associated with digital health literacy among stroke patients included gender (28, 29), the presence of comorbidities (21, 22, 26), Barthel Index (22, 28), type of medical insurance (25), self-rated health status (29), and cognitive function (33).

##### 3.3.1.2 Psychological and self-regulation characteristics

Self-efficacy (22, 24, 29, 34), perceived progress (34, 36), positive psychological capital (25), anxiety and depression (28), self-management ability (28), and health behavior levels (22) were identified as factors influencing digital health literacy among stroke patients. Four studies showed that self-efficacy was a significant influencing factor (22, 24, 29, 34), with higher self-efficacy being associated with higher levels of digital health literacy. Two studies reported a positive association between perceived progress and digital health literacy (34, 36), indicating that patients with a more favorable subjective evaluation of their own rehabilitation progress tended to have better digital health literacy. Patients with better psychological status, stronger self-management ability, and healthier behavioral patterns generally exhibited higher levels of digital health literacy. In contrast, negative emotions such as anxiety and depression may reduce patients’ willingness to seek information and their initiative in health management, thereby leading to lower levels of digital health literacy.

##### 3.3.1.3 Technology-related attitudes and digital health capability

Attitudes toward online health information (23, 29), willingness to receive information and communication technology services (e.g., telemedicine) (21, 32), health information–seeking ability (24), and self-perceived proficiency in using digital devices (22) were identified as factors associated with digital health literacy among stroke patients. Two studies suggested that stroke patients who held positive attitudes toward online health information had higher levels of digital health literacy (23, 29). Another two studies showed that patients who were willing to receive information and communication technology services, such as telemedicine, also tended to report higher levels of digital health literacy (21, 32). These findings suggest that technology-related attitudes, such as willingness to receive information and communication technology services, may influence stroke patients’ engagement with digital health information and services, whereas digital health capability may support their ability to access and use such resources; both were associated with digital health literacy.

#### 3.3.2 Environmental factors

Environmental factors included family and social support as well as the information and service environment.

##### 3.3.2.1 Family and social support

Social support was identified as a factor influencing digital health literacy among stroke patients. Two studies indicated that patients with better social support tended to have higher levels of digital health literacy (25, 29). Patients living with family members and receiving greater family support also exhibited higher levels of digital health literacy (29, 33, 36). Meanwhile, attributes of caregivers, including their digital health literacy and related capability levels, may also influence the digital health literacy of stroke patients (25, 30).

##### 3.3.2.2 Information and service environment

Four studies showed that perceived accessibility and convenience were important factors influencing digital health literacy among stroke patients (31, 34–36). In general, patients believed that digital health interventions could reduce the burden of travel and waiting time, provide more flexible service hours, and save time and effort, with particular advantages for those with mobility difficulties, those living in remote areas, or those with physical limitations. Continuous care, emotional support, and clear informational feedback also helped enhance patients’ sense of reassurance and satisfaction (31, 34). At the same time, technological barriers (31, 35, 36) and clinical limitations (31, 35) were important constraints. Difficulties with logging in, unstable internet connections, inadequate access to devices, limited operational skills, and difficulty adapting to new technologies all suggest that the digital divide remains present. In addition, the limitations of remote services in physical examination, observation of subtle symptoms, management of emergency situations, and support for complex communication further restricted their effectiveness. Other factors that may influence the development of digital health literacy among stroke patients include the credibility of online information (26), time arrangements (31, 36), the availability and type of internet-enabled devices and tools (26, 30), and whether patients have sources and access channels for obtaining health information online (21, 23, 26, 30).

#### 3.3.3 Behavioral factors

Behavioral factors comprised proactive information behaviors and interactive and communication behaviors.

##### 3.3.3.1 Proactive information behaviors

Six studies identified daily internet use duration as an important factor influencing digital health literacy among stroke patients (22, 24, 26–29). Patients who spent more time online each day tended to have higher levels of digital health literacy. Five studies further showed that the frequency of seeking health information online was also associated with digital health literacy (21, 23, 24, 29, 30), with patients who searched for online health information more frequently generally demonstrating higher levels of health literacy. In addition, experience in seeking health information online (27, 28), length of internet use (27), frequency of internet use (21, 26), and whether patients used the internet (30) were all related to digital health literacy among stroke patients. Patients with prior experience in seeking health information online, longer internet use duration, and more frequent internet use tended to have higher levels of digital health literacy.

##### 3.3.3.2 Interactive and communication behaviors

In terms of interactive and communication behaviors, the frequency of seeking help from others when using the internet (22), active dissemination of online health information (23), and willingness to interact with individuals with mental illness (21) were also important behavioral factors influencing digital health literacy among stroke patients. Patients with stroke who more frequently sought help from others while using the internet, actively shared online health knowledge, and were willing to interact with individuals with mental illness tended to have higher levels of digital health literacy.

## 4 Discussion

This study integrated and analyzed the factors influencing digital health literacy among stroke patients based on the triadic reciprocal model of SCT. The findings showed that digital health literacy in stroke patients is not determined by a single factor, but rather is dynamically shaped by the interaction among personal, environmental, and behavioral factors. From the perspective of SCT (15), these findings suggest that digital health literacy can be understood as the product of reciprocal influences among these three domains. Specifically, personal factors such as psychological readiness, cognitive capacity, and acceptance of digital technology may affect patients’ willingness and ability to engage in digital health activities. Environmental factors, including family support, caregiver assistance, and accessible digital health services, may further facilitate or hinder such engagement (37, 38). Through repeated information-seeking, communication, and use of digital health resources, patients may gradually strengthen their self-efficacy and digital skills, which in turn may promote further engagement and improvement in digital health literacy (39).

Personal factors constitute an important internal foundation influencing digital health literacy among stroke patients, and mainly encompass three domains: sociodemographic and health-related characteristics, psychological and self-regulation characteristics, and technology-related attitudes and digital health capability. Age is one of the most important determinants of digital health literacy. Older patients often demonstrate lower levels of digital health literacy, which may be attributable to age-related functional decline, reduced comprehension, slower adaptation to emerging technologies, and a more cautious or even avoidant attitude toward online information (40). A higher educational level is also associated with stronger abilities to identify, understand, and appraise information, and may promote more proactive engagement with digital health resources (41–43). Patients with better financial capacity usually have greater access to digital devices and more stable internet connectivity (44), which may in turn support sustained use of digital health services and facilitate the development of higher digital health literacy. In addition, place of residence and employment status may also influence digital health literacy among stroke patients. Urban residents generally benefit from more developed internet infrastructure, more diverse health care and information resources, and more convenient technical support (45), and accordingly tend to report higher levels of digital health literacy than rural residents. Employed patients appear to have higher digital health literacy than retired or unemployed patients, which may be related to their greater degree of social participation and more frequent exposure to digital devices in daily life. Differences across occupational categories further suggest that occupational groups with better social resources and information environments may be at an advantage in terms of digital health literacy. Notably, findings regarding marital status remain inconsistent. Some studies suggest that unmarried patients with stroke have higher levels of digital health literacy, whereas married and widowed patients tend to have lower levels. However, another study reported that patients with a spouse had higher digital health literacy. Having a spouse may provide stronger emotional and caregiving support, thereby facilitating access to and use of digital health resources. Conversely, it may also foster dependence on caregivers and reduce patients’ motivation to seek information and use digital resources independently. Patients with multiple comorbidities, poorer activities of daily living, cognitive impairment, and poorer self-rated health generally tend to have lower levels of digital health literacy. This may be because greater disease burden and functional limitations reduce patients’ capacity and motivation to engage in digital health behaviors, while also diminishing their efficiency in searching for, understanding, evaluating, and applying health information (46).

Beyond sociodemographic and health-related characteristics, patients’ psychological status and self-regulation capacity may also play an important role in the development of digital health literacy. Self-efficacy appears to be one of the most important psychological determinants. Patients with higher self-efficacy are generally more confident in their ability to learn and use digital health tools and are more willing to actively seek, understand, appraise, and apply online health information, thereby demonstrating higher levels of digital health literacy. Among stroke patients, post-stroke functional limitations, role transitions, and concerns about recurrence may undermine their sense of control (47), whereas stronger self-efficacy may help them maintain active engagement and persistence when facing challenges related to digital device operation, information filtering, and online health management (48). Perceived progress, positive psychological capital, self-management capacity, and health-promoting behaviors have also been positively associated with digital health literacy, suggesting that positive experiences during rehabilitation and stronger self-regulatory abilities may enhance patients’ willingness to engage with digital health resources and sustain their use over time. In contrast, negative emotional states such as anxiety and depression may weaken patients’ motivation to obtain health information and reduce their initiative in health management, thereby hindering the development of digital health literacy.

Technology-related attitudes and digital health capability are also important personal factors associated with digital health literacy. Attitudes toward online health information may shape patients’ willingness to engage with digital health resources. Patients with more positive attitudes are generally more likely to actively seek relevant information and, through repeated exposure, gradually enhance their ability to understand and appraise online health content (49, 50). Similarly, willingness to accept information and communication technology–based services may influence patients’ readiness to try and use digital services such as telemedicine and mobile health. Those with stronger willingness are more likely to enter digital health settings, thereby gaining greater opportunities for learning and application. Health information–seeking ability supports the development of the development of digital health literacy, as it directly affects patients’ capacity to obtain, filter, and use online health information effectively (46). Perceived proficiency in using digital devices also reflects patients’ operational skills with digital tools. Greater proficiency may reduce technical barriers and improve the efficiency of using digital health information and services. Overall, personal factors may influence digital health literacy through different but interrelated pathways. Sociodemographic and health-related characteristics provide the basic foundation, psychological and self-regulation characteristics shape motivation and persistence, and technology acceptance and digital health capability affect patients’ readiness to engage with digital health resources. Together, these factors help explain individual differences in the development of digital health literacy among stroke patients.

Environmental factors may serve as important external conditions influencing whether digital health literacy can be translated into practical capability among stroke patients. Family and social support appear to be particularly important in this process. Support from family members, friends, and healthcare professionals may provide informational guidance, emotional encouragement, and practical assistance, thereby facilitating patients’ ability to access, identify, and use online health information effectively (51). This may be especially relevant for stroke patients, who often experience physical, cognitive, or language impairments after stroke. In such cases, caregivers frequently play a crucial supportive role in digital device use, information filtering, and health-related decision-making (52), while caregivers’ own competence and digital health literacy may further shape patients’ access to digital health resources (53). The information and service environment may also influence the development of digital health literacy. In this study, perceived accessibility, convenience, and continuous feedback were identified as important facilitators. Patients generally perceived that digital health services could reduce travel, waiting time, and physical burden, offer greater flexibility, and improve healthcare access for those living in remote areas or experiencing mobility limitations (31, 34–36). These advantages may not only enhance the patient experience but also strengthen patients’ willingness and confidence to engage in digital health services. At the same time, continuity of care, timely feedback, and emotional support may promote a greater sense of security and satisfaction, thereby encouraging sustained participation in digital health activities. From a social cognitive perspective (15), such positive environmental feedback may reinforce health behaviors and support the continued development of digital health literacy. However, environmental factors may also hinder the development of digital health literacy. Difficulties with logging in, unstable internet connections, limited device availability, complex operations, and poor adaptation to new technologies suggest that a digital divide remains common among stroke patients, particularly among older adults, rural residents, and those with functional impairments. In addition, limitations of remote services in physical examination, subtle symptom recognition, emergency response, and complex communication may reduce patients’ trust in digital care and lead some to prefer face-to-face services (35). The perceived credibility of online information, access to internet-enabled devices, and the sources and channels of health information may also affect patients’ willingness to engage with and use digital health resources. Although these studies did not always examine digital health literacy directly, their findings remain relevant because they show how environmental factors may shape stroke patients’ opportunities and practical conditions for engaging with digital health information and services. Overall, environmental factors may influence digital health literacy through related but distinct pathways. Family and social support may provide informational, emotional, and practical assistance that facilitates patients’ engagement with digital health resources, whereas the information and service environment may affect whether such resources are accessible, understandable, and usable in everyday care. Together, these factors shape the external context in which stroke patients use digital health resources and services.

Behavioral factors may represent important behavioral correlates of digital health literacy among stroke patients. In terms of proactive information behaviors, this study found that patients who spent more time online each day, used the internet more frequently, had longer internet use experience, and had prior experience searching for online health information or searching for health information more regularly tended to show higher levels of digital health literacy. However, these associations should be interpreted cautiously, because internet use and online health information seeking may function both as factors associated with the development of digital health literacy and as behaviors that are more likely to occur among patients who already have higher digital health literacy. Frequent internet use and health information seeking may help patients become more familiar with operating digital devices, increase their exposure to health resources (43), and enhance health awareness, attitudes, and behaviors through the repeated process of retrieving, understanding, and applying information (54), which may in turn support the development of digital health literacy. At the same time, patients with higher digital health literacy may also be more capable of and more inclined to engage in frequent internet use and online health information seeking. Digital health literacy should not be viewed as a static body of knowledge, but rather as a dynamic capability that develops through repeated exposure to, searching for, appraising, communicating, and applying digital information (55). In this sense, behavior may be both an outcome of digital health literacy and an important pathway through which it continues to develop. Interactive and communicative behaviors may also play an important role. Patients who more frequently sought help from others during internet use or actively shared online health information tended to have higher levels of digital health literacy. Seeking help from family members, peers, or healthcare professionals may provide technical guidance, informational support, and emotional encouragement, thereby reducing frustration during digital engagement and strengthening patients’ confidence in participating in digital health activities (52, 56). Active dissemination of health information may, to some extent, reflect patients’ ability to understand, endorse, and reprocess health information. Such behaviors may not only reinforce patients’ own mastery of health information but also further contribute to the consolidation of digital health literacy. Overall, behavioral factors may be closely associated with digital health literacy through different but related pathways. Proactive information behaviors may increase patients’ opportunities to access, understand, and apply digital health information, whereas interactive and communicative behaviors may strengthen their confidence, reinforce understanding, and support continued engagement in digital health activities. Together, these behaviors may be closely associated with how stroke patients develop and sustain digital health literacy in practice.

These findings have several implications for clinical practice and rehabilitation in stroke care. For stroke survivors at risk of limited digital health literacy, early screening should extend beyond sociodemographic profiling to include post-stroke impairments that may directly hinder the use of digital health services, such as cognitive fatigue, slowed information processing, aphasia, visual field deficits or visuospatial neglect, and upper-limb motor impairment. These deficits may compromise not only patients’ ability to seek, understand, and appraise online health information, but also their capacity to perform routine digital tasks, including logging into accounts, navigating multi-step interfaces, typing, interpreting icons, and maintaining attention during telehealth encounters. Accordingly, alongside health education aimed at improving patients’ ability to access, understand, evaluate, and use digital health tools and online health information, as well as strengthening their motivation and self-efficacy, stroke-specific supportive strategies are also needed to enhance the accessibility and usability of digital health services. These may include simplified, low-burden workflows; plain-language content adapted for people with aphasia and supported by multimodal cues; high-contrast, clearly structured, and uncluttered interfaces for those with visual deficits; and larger touch targets or voice-input options for those with motor impairment. In addition, caregiver involvement should be regarded as a central component of stroke-specific digital health support. Many stroke survivors require assistance with device operation, information interpretation, and healthcare-related decision-making, particularly those with aphasia, cognitive impairment, or physical disability. Therefore, integrating caregivers into digital health education and skills training, enabling proxy access or shared-device functions, and providing ongoing technical support throughout rehabilitation may help patients overcome stroke-related barriers and sustain long-term engagement with digital health services.

This study still has several limitations. First, the geographical distribution of the included studies was relatively concentrated, with most studies conducted in China. Therefore, caution is needed when generalizing the findings across different cultural contexts. This issue is particularly relevant to the interpretation of environmental factors, especially support-related factors. Although social support was assessed in the included studies as a broad construct, the observed role of support may have been shaped by the Chinese post-stroke care context, in which family members and informal caregivers often play a central role. As a result, the environmental findings identified in this review may more closely reflect family-centered caregiving settings and may not be fully transferable to contexts where support is delivered primarily through formal healthcare services or community-based systems. Second, the included evidence was dominated by quantitative studies, whereas qualitative studies were relatively limited and mixed-methods research was lacking, which may have restricted a more in-depth understanding of the factors influencing digital health literacy among stroke patients. Third, substantial heterogeneity was observed across the included studies in terms of digital health literacy measurement tools, definitions of influencing factors, and participant characteristics. Some studies used general instruments such as e-Health Literacy Scale (eHEALS) and eHealth Literacy Questionnaire (eHLQ), whereas others employed self-developed questionnaires specifically designed for stroke patients. Because these instruments capture somewhat different dimensions of digital health literacy, this heterogeneity limited the comparability of findings across studies and prevented a standardized comparison of baseline digital health literacy across different international cohorts. In addition, the included populations varied in age, stage of disease, and health status, which increased the complexity of cross-study comparison and evidence synthesis. These limitations highlight the need for the development and validation of a standardized, stroke-specific digital health literacy assessment tool in future research. Moreover, all quantitative studies included in this review were cross-sectional. Therefore, although several behavioral variables, such as internet use and online health information seeking, were associated with digital health literacy, the direction of these associations remains uncertain, and causal relationships cannot be established. Fourth, although SCT offered a useful framework for classifying the factors identified in this review, it may be less precise in capturing technology-related influences. In particular, factors such as device availability, internet accessibility, and the usability of digital health services were categorized within the environmental domain, as they represent external conditions that may facilitate or constrain patients’ use of digital health resources. Although this categorization is theoretically consistent with SCT, the framework may be less precise than technology-oriented frameworks in explaining these technological and structural influences. This limitation highlights the value of integrating SCT with technology-oriented or sociotechnical frameworks to better understand how digital health environments shape digital health literacy among stroke patients.

## 5 Conclusion

This study systematically synthesized the multidimensional factors influencing digital health literacy among stroke patients and demonstrated that it is shaped by the dynamic interaction of personal, environmental, and behavioral factors. In clinical practice, healthcare professionals should pay particular attention to digitally vulnerable groups and develop targeted interventions based on patients’ individual characteristics and digital health needs, including improving digital skills, enhancing confidence in technology use, strengthening social support, and increasing the accessibility of digital health services. Further high-quality research is needed to expand the evidence base on factors influencing digital health literacy among stroke patients and to inform the development of precise intervention strategies and improved rehabilitation management.

## Data Availability

The original contributions presented in the study are included in the article/Supplementary material, further inquiries can be directed to the corresponding author.
