# Supplementary material for: Factors influencing digital health literacy in stroke patients based on social cognitive theory: a mixed-methods systematic review
Source: Front Public Health. 2026 Aug 17;14:1905179. doi: 10.3389/fpubh.2026.1905179 (PMC13526950; doi:10.3389/fpubh.2026.1905179)
Supplement: Supplementary file 1 [file Supplementary_file_1.docx]

Supplementary Material

# TABLE S1 The detailed PubMed search strategy

| Number | Search terms |
| --- | --- |
| #1 | “Stroke”[Mesh] OR "Ischemic Stroke"[Mesh] OR "Hemorrhagic Stroke"[Mesh] |
| #2 | stroke[Title/Abstract] OR "cerebral stroke"[Title/Abstract] OR cerebrovascular stroke*[Title/Abstract] OR cerebrovascular accident*[Title/Abstract] OR CVA[Title/Abstract] OR "cerebrovascular event"[Title/Abstract] OR "cerebrovascular infarction"[Title/Abstract] OR "cerebrovascular apoplexy"[Title/Abstract] OR apoplexy[Title/Abstract] OR brain vascular accident*[Title/Abstract] OR "ischemic stroke"[Title/Abstract] OR "ischaemic stroke"[Title/Abstract] OR cerebral infarct*[Title/Abstract] OR cerebral thrombo*[Title/Abstract] OR cerebral emboli*[Title/Abstract] OR "brain ischemia"[Title/Abstract] OR brain infarct*[Title/Abstract] OR brain thrombo*[Title/Abstract] OR brain emboli*[Title/Abstract] OR "hemorrhagic stroke"[Title/Abstract] OR "haemorrhagic stroke"[Title/Abstract] OR "intracerebral hemorrhage"[Title/Abstract] OR "cerebral hemorrhage"[Title/Abstract] OR "subarachnoid hemorrhage"[Title/Abstract] OR "brain hemorrhage"[Title/Abstract] OR "lacunar infarct"[Title/Abstract] |
| #3 | #1 OR #2 |
| #4 | “Digital Health”[Mesh] |
| #5 | "digital health"[Title/Abstract] OR "digital health literacy"[Title/Abstract] OR "ehealth literacy"[Title/Abstract] OR "e-health literacy"[Title/Abstract] OR "electronic health literacy"[Title/Abstract] OR "online health literacy"[Title/Abstract] OR "digital literacy"[Title/Abstract] OR "mobile health literacy"[Title/Abstract] |
| #6 | #4 OR #5 |
| #7 | "Influencing factor"[Title/Abstract] OR determinant[Title/Abstract] OR factor*[Title/Abstract] OR facilitator*[Title/Abstract] OR barrier*[Title/Abstract] OR enabler*[Title/Abstract] OR motivator*[Title/Abstract] OR implementation[Title/Abstract] OR support[Title/Abstract] OR experience*[Title/Abstract] OR perspective*[Title/Abstract] OR perception*[Title/Abstract] OR attendance[Title/Abstract] OR predictor*[Title/Abstract] OR preference[Title/Abstract] OR feedback*[Title/Abstract] |
| #8 | #3 AND #6 AND #7 |

# TABLE S2 Quality assessment according to Mixed Methods Appraisal Tool (MMAT)

| **Qualitative studies** | | | | | | | | **MMAT criteria met (n/5)** |
| --- | --- | --- | --- | --- | --- | --- | --- | --- |
| Author (Year) | **Screening questions** | | **Methodological quality criteria** | | | | |  |
|  | S1. Are there clear research questions? | S2. Do the collected data allow to address the research questions? | 1.1. Is the qualitative approach appropriate to answer the research question? | 1.2. Are the qualitative data collection methods adequate to address the research question? | 1.3. Are the findings adequately derived from the data? | 1.4. Is the interpretation of results sufficiently substantiated by data? | 1.5. Is there coherence between qualitative data sources, collection, analysis and interpretation? |  |
| Wong AKC (31) (2025) | Y | Y | Y | Y | Y | Y | Y | 5/5 |
| Gaboury I (34) (2022) | Y | Y | Y | Y | Y | Y | Y | 5/5 |
| Chan S (35) (2021) | Y | Y | Y | Y | Y | Y | Y | 5/5 |
| Chen Y (36) (2020) | Y | Y | Y | Y | Y | Y | U | 4/5 |
| **Quantitative studies** | | | | | | | | **MMAT criteria met (n/5)** |
| Author (Year) | **Screening questions** | | **Methodological quality criteria** | | | | |  |
|  | S1. Are there clear research questions? | S2. Do the collected data allow to address the research questions? | 3.1. Are the participants representative of the target population? | 3.2. Are measurements appropriate regarding both the outcome and intervention (or exposure)? | 3.3. Are there complete outcome data? | 3.4. Are the confounders accounted for in the design and analysis? | 3.5. During the study period, is the intervention administered (or exposure occurred) as intended? |  |
| Cho M-K (32) (2025) | Y | Y | Y | Y | Y | N | Y | 4/5 |
| Linnestad AM (33) (2025) | Y | Y | Y | Y | Y | Y | Y | 5/5 |
| Xue M (21) (2025) | Y | Y | Y | Y | Y | Y | Y | 5/5 |
| Zhang C (22) (2025) | Y | Y | Y | Y | Y | Y | Y | 5/5 |
| Duan Y (23) (2024) | Y | Y | Y | Y | Y | Y | Y | 5/5 |
| Hu Y (24) (2024) | Y | Y | Y | Y | Y | Y | Y | 5/5 |
| Zhang J (25) (2024) | Y | Y | Y | Y | Y | Y | Y | 5/5 |
| Liu B (26) (2023) | Y | Y | Y | Y | Y | Y | Y | 5/5 |
| Wang X (27) (2023) | Y | Y | Y | Y | Y | Y | Y | 5/5 |
| Yu J (28) (2023) | Y | Y | Y | Y | Y | Y | Y | 5/5 |
| He Y (29) (2022) | Y | Y | Y | Y | Y | Y | Y | 5/5 |
| Zhang Z (30) (2021) | Y | Y | Y | Y | Y | Y | Y | 5/5 |

Y=Yes, N=No, U=Unclear

# TABLE S3 Factors derived from included studies

| **SCT component** | **Integrated theme** | **Factor** | **Evidence source** | **Original findings** | **Qualitized statement** |
| --- | --- | --- | --- | --- | --- |
| Personal factors | Sociodemographic and health-related characteristics | Age | Quantitative | Multiple regression analysis indicated that age ... influenced the level of digital health literacy (*p* < 0.05) (22) Binary logistic regression showed that stroke patients aged over 60 years were less likely to have a high level of eHealth literacy than those aged 18–44 years (OR = 0.388, 95% CI: 0.151–0.996, *p* = 0.049) (23) There were statistical differences in electronic health literacy scores of acute ischemic stroke patients with different ...age... (*p* < 0.05) (25) eHealth literacy scores differed significantly by age,... (*p* < 0.05) (27) Multivariate linear regression showed that age, ...were included in the regression equation, and the differences were statistically significant (*p* < 0.001) (28) Across different latent eHealth literacy classes, stroke patients showed statistically significant differences in the distribution of age, ... (29) Binary Logistic regression analysis showed that age of stroke inpatients was associated with the level of eHealth literacy of stroke inpatients（*p* < 0.05）(30) Participants who are older, .. are more likely to be a Novice and less likely to be a Cautious user (33) | Older age appeared to be associated with lower digital health literacy among stroke patients, with younger patients tending to have higher digital health literacy and more favorable literacy profiles. |
|  |  | Educational level | Quantitative | Compared with the low e-health literacy group, participants with lower levels of education were less likely to enter the high literacy-low decision-making group (all *p* < 0.05) (21) There were significant differences in digital health literacy by ... education level,..(*p* < 0.05) (22) Stroke patients with a college degree or above were 3.824 times more likely to have a high level of eHealth literacy than those with only primary school education (OR = 3.824, 95% CI: 1.276–11.459, *p* = 0.017) (23) Education level was entered into the regression equation (*p* < 0.05) (24) There were statistical differences in electronic health literacy scores of acute ischemic stroke patients with different educational level,... (*p* < 0.05) (25) Multiple linear regression analysis showed that ...education level were the independent influencing factors of ehealth literacy of stroke patients (*p* < 0.05) (26) Among young and middle-aged stroke patients, eHealth literacy scores differed significantly by ... education level, ...(*p* < 0.05) (27) Multivariate linear regression showed that...educational level,...were included in the regression equation, and the differences were statistically significant (*p* < 0.001) (28) Across different latent eHealth literacy classes, stroke patients showed statistically significant differences in the distribution of ...education degree... (29) There were statistically significant differences in the eHealth literacy pass rate among hospitalized stroke patients of different...education levels,... (*p* < 0.05) (30) Participants who have a lower level of education are more likely to be a Novice and less likely to be a Cautious user (33) | Higher educational attainment appeared to be associated with higher digital health literacy among stroke patients, with better-educated patients more likely to belong to more favorable literacy profiles. |
|  |  | Financial status | Quantitative | There were significant differences in digital health literacy by...personal monthly income... (*p* < 0.05) (22) There were statistically significant differences in eHealth literacy scores by ... income... (*p* < 0.05) (23) There were statistical differences in electronic health literacy scores of acute ischemic stroke patients with different ...monthly income... (*p* < 0.05) (25) There were significant differences in eHealth literacy levels among stroke patients by...personal monthly income ... (*p* < 0.05) (26) Among young and middle-aged stroke patients, eHealth literacy scores differed significantly by ...monthly income, ... (*p* < 0.05) (27) There were statistically significant differences in eHealth literacy scores among patients of different ...per capita monthly household income... (*p* < 0.05) (28) Across different latent eHealth literacy classes, stroke patients showed statistically significant differences in the distribution of ... income..(29) There were statistically significant differences in the eHealth literacy pass rate among hospitalized stroke patients of different ...average monthly household income per capita...(*p* < 0.05) (30) | Higher monthly income appeared to be associated with higher digital health literacy among stroke patients, with greater financial resources linked to more favorable literacy profiles. |
|  |  | Residence | Quantitative | There were significant differences in digital health literacy by...current place of residence...(*p* < 0.05) (22) There were statistically significant differences in eHealth literacy scores by ... residence... (*p* < 0.05) (23) There were significant differences in eHealth literacy among patients with different...places of residence... (*p* < 0.05) (24) There were statistical differences in electronic health literacy scores of acute ischemic stroke patients with different... residence (*p* < 0.05) (25) Across different latent eHealth literacy classes, stroke patients showed statistically significant differences in the distribution of ...place of residence... (29) | Urban residence appeared to be associated with higher digital health literacy among stroke patients, whereas rural residence tended to be associated with lower digital health literacy and less favorable literacy profiles. |
|  |  | Employment status | Quantitative | There were significant differences in digital health literacy by ...employment status... (*p* < 0.05) (22) There were statistical differences in electronic health literacy scores of acute ischemic stroke patients with different ...working status (*p* < 0.05) (25) There were statistically significant differences in eHealth literacy scores among patients of different ...employment statuses,... (*p* < 0.05) (28)  The proportion not in paid work was highest in the Novices group [91% (42/46)], followed by Cautious users [66% (66/101)] and Navigators [47% (14/30)], with a statistically significant between-group difference (*p* < 0.001) (33) | Employment status appeared to be associated with digital health literacy among stroke patients, with employed patients tending to have higher digital health literacy than retired, unemployed, or unpaid patients. |
|  |  | Occupation type | Quantitative | There were significant differences in eHealth literacy among patients with different ...occupations... (*p* < 0.05) (24) Among young and middle-aged stroke patients, eHealth literacy scores differed significantly by ...occupation... (*p* < 0.05) (27) There were statistically significant differences in the eHealth literacy pass rate among hospitalized stroke patients of different...occupations... (*p* < 0.05) (30) | Occupation type appeared to be associated with digital health literacy among stroke patients, with those in more skilled or non-manual occupations tending to have higher digital health literacy, whereas farmers and manual workers were more likely to have lower digital health literacy. |
|  |  | Marriage status | Quantitative | Among young and middle-aged stroke patients, eHealth literacy scores differed significantly by... marital status, ... (*p* < 0.05) (27) Across different latent eHealth literacy classes, stroke patients showed statistically significant differences in the distribution of ... spouse... (29) | Marital status appeared to be associated with digital health literacy among stroke patients, although the direction of this association varied across studies. |
|  |  | Gender | Quantitative | There were statistically significant differences in eHealth literacy scores among patients of different genders... (*p* < 0.05) (28) Across different latent eHealth literacy classes, stroke patients showed statistically significant differences in the distribution of ...gender... (29) | Gender appeared to be associated with digital health literacy among stroke patients, with male patients more likely to have higher digital health literacy and more favorable literacy profiles. |
|  |  | Comorbidities | Quantitative | Compared with the low e-health literacy group, participants without hyperlipidaemia ... were more likely to enter the low application-high decision-making group and high literacy-low decision-making group (*p* < 0.05) (21) There were significant differences in digital health literacy by...number of underlying diseases... (*p* < 0.05) (22) There were significant differences in eHealth literacy levels among stroke patients by ...whether suffering from hypertension... (*p* < 0.05) (26) | The presence of comorbidities appeared to be associated with lower digital health literacy among stroke patients, with those without comorbidities more likely to have higher digital health literacy and more favorable literacy profiles. |
|  |  | Barthel Index | Quantitative | Multiple regression analysis indicated that ... Barthel Index...significantly influenced the level of digital health literacy (*p* < 0.05) (22) It was positively correlated with the score of BI (*r* = 0.710) (28) | Higher Barthel Index scores appeared to be associated with higher digital health literacy among stroke patients. |
|  |  | Type of medical insurance | Quantitative | There were statistical differences in electronic health literacy scores of acute ischemic stroke patients with different ... medical expense type... (*p* < 0.05) (25) | Type of medical insurance appeared to be associated with digital health literacy among stroke patients, with employee medical insurance tending to be associated with higher digital health literacy than urban–rural resident medical insurance. |
|  |  | Self-rated health status | Quantitative | Across different latent eHealth literacy classes, stroke patients showed statistically significant differences in the distribution ...self-rated health status... (29) | Better self-rated health status appeared to be associated with higher digital health literacy among stroke patients and with membership in more favorable literacy profiles. |
|  |  | Cognitive function | Quantitative | Participants who have a lower level of cognitive function are more likely to be a Novice and less likely to be a Cautious user (33) | Higher cognitive function appeared to be associated with higher digital health literacy among stroke patients, with lower cognitive function linked to less favorable literacy profiles. |
|  | Psychological and self-regulation characteristics | Self-efficacy | Quantitative and Qualitative (corroboration) | Multiple regression analysis indicated that... self-efficacy significantly influenced the level of digital health literacy (*p* < 0.05) (22) Information self-efficacy was positively correlated with eHealth literacy (*p* < 0.05) (24) Self-efficacy scores also differed significantly among the different latent eHealth literacy classes (*p* < 0.05) (29) "even though patients were intimidated by the technology at first, they all reported being confident about the use of the platform because it was simple to use" (34) | Self-efficacy appeared to be positively associated with digital health literacy among stroke patients, with higher self-efficacy linked to higher digital health literacy and more favorable digital health literacy profiles. |
|  |  | Perceived progress | Qualitative | "Every patient perceived improvement in their function and reported reaching their personal goals" (34) "my arm started getting a little stronger I could reach more you know in and I practiced I started reaching for the refrigerator with my right hand and door knobs". "participants were motivated when they subjectively experienced progress or their caregivers observed the progress" (36) | / |
|  |  | Positive psychological capital | Quantitative | The total score of electronic health literacy was also positively associated with positive psychological capital, the correlation coefficient was 0.492, *p* < 0.01 (25) | Positive psychological capital appeared to be positively associated with digital health literacy among stroke patients. |
|  |  | Anxiety and depression | Quantitative | Multivariate linear regression showed that ... anxiety, depression ... were included in the regression equation, and the differences were statistically significant (*p* < 0.001) (28) | Anxiety and depression appeared to be negatively associated with digital health literacy among stroke patients, with higher levels of anxiety and depression linked to lower digital health literacy. |
|  |  | Self-management ability | Quantitative | Multivariate linear regression showed that... self-management ability were included in the regression equation, and the differences were statistically significant (*p* < 0.001) (28) | Self-management ability appeared to be positively associated with digital health literacy among stroke patients, with stronger self-management linked to higher digital health literacy. |
|  |  | Health behavior level | Quantitative | Multiple regression analysis indicated that... health behaviors...significantly influenced the level of digital health literacy (*p* < 0.05) (22) | Health behavior level appeared to be positively associated with digital health literacy among stroke patients, with better health behaviors linked to higher digital health literacy. |
|  | Technology acceptance and digital health capability | Attitudes toward online health information | Quantitative | Patients with a neutral attitude toward online health information were 3.431 times more likely to have a high level of eHealth literacy than those who did not trust online health information (OR = 3.431, 95% CI: 1.421–8.285, *p* = 0.006) (23) Across different latent eHealth literacy classes, stroke patients showed statistically significant differences in the distribution of ...attitude of online health information... (29) | More positive attitudes toward online health information appeared to be associated with higher digital health literacy among stroke patients, with greater trust in and perceived usefulness of online health information linked to more favorable digital health literacy profiles. |
|  |  | Willingness to receive ICT services | Quantitative | Compared with the low e-health literacy group, participants who were unwilling to receive remote care (OR = 2.382, *p* = 0.003) were more likely to enter the low application-high decision-making group (21) The mobile health literacy of the group willing to use ICT services was significantly higher than that of the group unwilling (*t* = 4.11, *p* < 0.001) (32) | Willingness to receive ICT services appeared to be associated with digital health literacy among stroke patients, with greater willingness linked to higher digital health literacy, whereas unwillingness tended to be associated with less favorable literacy-related profiles. |
|  |  | Health information–seeking ability | Quantitative | Health information seeking was positively correlated with eHealth literacy (*p* < 0.05) (24) | Health information seeking appeared to be positively associated with digital health literacy among stroke patients, with stronger health information-seeking ability linked to higher digital health literacy. |
|  |  | Self-perceived digital device use proficiency | Quantitative | Multiple regression analysis indicated that ...self-perceived proficiency in using digital devices...significantly influenced the level of digital health literacy (all *p* < 0.05) (22) | Higher self-perceived proficiency in using digital devices appeared to be associated with higher digital health literacy among stroke patients. |
| Environmental factors | Family and social support | Social support | Quantitative | There was a significant positive association with social support, the correlation coefficient was 0.460, *p* < 0.01 (25) Social support differed significantly among the different latent eHealth literacy classes (*p* < 0.05) (29) | Higher social support appeared to be associated with higher digital health literacy among stroke patients, with greater social support linked to more favorable digital health literacy profiles. |
|  |  | Family support | Quantitative and Qualitative (corroboration) | Across different latent eHealth literacy classes, stroke patients showed statistically significant differences in the distribution of ...whether living with family... (29) Those who live alone are significantly less likely to be a Navigator and more likely to be a Cautious user (33) "I have a son who comes in and out of the house about once a week and he was thrilled over anything that I was getting to help" (36) | Family support appeared to be associated with higher digital health literacy among stroke patients, with living with family linked to more favorable digital health literacy profiles. |
|  |  | Caregiver level | Quantitative | There were statistical differences in electronic health literacy scores of acute ischemic stroke patients with different ...primary caregivers level... (*p* < 0.05) (25) Binary Logistic regression analysis showed that eHealth literacy of their caregivers was associated with the level of eHealth literacy of stroke inpatients (*p* < 0.05) (30) | More favorable caregiver-level characteristics, including adequate caregiver support and higher caregiver digital health literacy, appeared to be associated with higher digital health literacy among stroke patients. |
|  | Information and service environment | Perceived accessibility and convenience | Qualitative | "Stroke patients often face mobility challenges, as their limbs may not be as agile, and some may still require the use of a cane. Traveling long distances to the hospital can be difficult for them". "Hospitals are extremely crowded nowadays. With teleconsultation, I don’t have to go to the hospital as often, and I can conveniently receive medical advice from home" (31)  "The biggest advantage for the patients was the time spared by not needing to commute" (34) "I didn’t have to come in [to the hospital] and I could understand [the doctor] better and it was on time. I knew that 10:30 was 10:30 when he was gonna be there. So it’s all positive as far as I’m concerned. It couldn’t be much better than that, you know. I mean, if there’s any questions I have, I can ask them same as if I’m standing in front of him. And I don’t have to be examined which is a plus for my case". "I had a heart attack after the stroke, so if I had to drive to Kingston, I’d be exhausted when I got there. This way, it was not demanding on me physically because all I had to do was sit in the chair. I didn’t have to drive all the way to Kingston" (35) "it was very convenient. You could go over there in your robe or pyjamas and do it if you didn’t want to get up at 8 o’clock in the morning" (36) | / |
|  |  | Supportive service experience | Qualitative | "I feel more relaxed during teleconsultation, maybe because I’m at home. The nurse was very caring and followed up closely, which helped relieve my stress". "When I’m at home on a video call, the nurse carefully asks me about what I have been eating.... I became more aware I should monitor my blood pressure regularly" (31) "some patients reported being worried that by not being in a rehabilitation center they would be less supported, but then all reported that telerehabilitation provided the opposite. One patient felt psychologically and physically supported. Another patient said he preferred at home interventions because his disabilities were not exposed to everyone in the therapy room" (34) | / |
|  |  | Technological barriers | Qualitative | "I downloaded the HA Go [the app used in Hong Kong for teleconsultation], but I wasn’t sure how to use it.... We ended up switching to a voice call" (31)  "I’m really not into electronics [laughs]. That’s because I don’t have a cell phone, you know. I’m not used to doing that. I just have an ordinary phone" "Um, it [in-person visit] probably would have triggered my mind to have asked about so and so, but because I wasn’t used to the computers, it was a little strange" (35) "the camera used for video-conferencing occasionally fell, which made them frustrated" (36) | / |
|  |  | Clinical limitations | Qualitative | "Through teleconsultation, nurses can only see the patient’s face.... But when assessing a stroke patient, being able to observe their movements and body language is crucial". "Some patients have slurred or distorted speech.... For more severe cases, effective conversation becomes nearly impossible" (31) "I would’ve probably found some satisfaction of going back to the hospital to have the visit because hospitals, you feel they know what they’re doing. Just because the hospital gives you that secure feeling. You think they know what they’re talking about" (35) | / |
|  |  | Credibility of online information | Quantitative | Multiple linear regression analysis showed that...Internet information credibility...were the independent influencing factors of ehealth literacy of stroke patients (*p* < 0.05) (26) | Higher perceived credibility of online health information appeared to be associated with higher digital health literacy among stroke patients. |
|  |  | Time arrangements | Qualitative | "Yes, it’s all during office hours.... I simply can’t answer calls during the day" (31) "he had to suspend some daily tasks if he was going to use the telerehabilitation system for six days a week. He wished for a less intense schedule, such as two days a week" (36) | / |
|  |  | Availability of internet-enabled devices and tools | Quantitative | There were significant differences in eHealth literacy levels among stroke patients by ...types of Internet access tools, ... (*p* < 0.05) (26) There were statistically significant differences in the eHealth literacy pass rate among hospitalized stroke patients of different ...vailability of internet access devices... (*p* < 0.05) (30) | More favorable internet access conditions, including having internet-enabled devices and using a wider range of internet access tools, appeared to be associated with higher digital health literacy among stroke patients. |
|  |  | Access to online health information sources and channels | Quantitative | Compared with the low e-health literacy group, participants who did not learn health information through family/friends (OR = 0.394, *p* = 0.004) were less likely to enter the low application-high decision-making group (21) There were statistically significant differences in eHealth literacy scores by ... access methods... (*p* < 0.05) (23) There were significant differences in eHealth literacy levels among stroke patients by ...number of online access to health information,... (*p* < 0.05) (26) There were statistically significant differences in the eHealth literacy pass rate among hospitalized stroke patients of different...sources of health information (accessing the internet via computer or mobile phone, etc.),...(*p* < 0.05) (30) | Greater access to online health information through multiple sources and channels appeared to be associated with higher digital health literacy among stroke patients, whereas limited or no access tended to be associated with lower digital health literacy. |
| Behavioral factors | Proactive information behaviors | Daily internet use duration | Quantitative | Multiple regression analysis indicated that ...daily internet use time...significantly influenced the level of digital health literacy (*p* < 0.05) (22) There were significant differences in eHealth literacy among patients with different ...daily time spent online... (*p* < 0.05) (24) There were significant differences in eHealth literacy levels among stroke patients by ...Internet access duration...(*p* < 0.05) (26) Stepwise multiple linear regression analysis showed that three variables were finally included in the regression equation. In ascending order of effect size, these were ... daily online time ... (27) There were statistically significant differences in eHealth literacy scores among patients of different ...average daily internet use time... (*p* < 0.05) (28) Across different latent eHealth literacy classes, stroke patients showed statistically significant differences in the distribution of ...online time (29) | Longer daily internet use duration appeared to be associated with higher digital health literacy among stroke patients. |
|  |  | Frequency of seeking health information online | Quantitative | Compared with the low e-health literacy group, participants with more frequent health inquiries were more likely to enter the high literacy-low decision-making group (all *p* < 0.05) (21)  Patients who frequently searched for online health information were 3.520 times more likely to have a high level of eHealth literacy than those who rarely searched for such information (OR = 3.520, 95% CI: 1.786–6.936, *p* < 0.001) (23) ...frequency of seeking health information...were entered into the regression equation (24) Across different latent eHealth literacy classes, stroke patients showed statistically significant differences in the distribution of...frequency of online health information search...(29) There were statistically significant differences in the eHealth literacy pass rate among hospitalized stroke patients of different ... frequency of searching for health-related information online... (*p* < 0.05) (30) | More frequent seeking of health information online appeared to be associated with higher digital health literacy among stroke patients. |
|  |  | Experience in seeking health information online | Quantitative | Stepwise multiple linear regression analysis showed that three variables were finally included in the regression equation. In ascending order of effect size, these were ...online searching for health information experience (27) Multivariate linear regression showed that ...experience of searching health information online...were included in the regression equation, and the differences were statistically significant (*p* < 0.001) (28) | Experience in seeking health information online appeared to be associated with higher digital health literacy among stroke patients. |
|  |  | Internet use history | Quantitative | Stepwise multiple linear regression analysis showed that three variables were finally included in the regression equation. In ascending order of effect size, these were Internet usage time... (27) | Longer internet use history appeared to be associated with higher digital health literacy among stroke patients. |
|  |  | Internet use frequency | Quantitative | Compared with the low e-health literacy group, ... more frequent Internet access were more likely to enter the low application-high decision-making group and high literacy-low decision-making group (both *p* < 0.05) (21) Multiple linear regression analysis showed that Internet access frequency...were the independent influencing factors of ehealth literacy of stroke patients (*p* < 0.05) (26) | Higher internet use frequency appeared to be associated with higher digital health literacy among stroke patients. |
|  |  | Internet use | Quantitative | There were statistically significant differences in the eHealth literacy pass rate among hospitalized stroke patients of different ...internet use status... (*p* < 0.05) (30) | Internet users tended to have higher digital health literacy than non-users. |
|  | Interactive and communication behaviors | Help-seeking when using the internet | Quantitative | Multiple regression analysis indicated that ...frequency of seeking help when using the internet...significantly influenced the level of digital health literacy (*p* < 0.05) (22) | More frequent help-seeking when using the internet appeared to be associated with higher digital health literacy among stroke patients. |
|  |  | Active dissemination of online health information | Quantitative | Patients who actively shared online health information were 2.311 times more likely to have a high level of eHealth literacy than those who did not (OR = 2.311, 95% CI: 1.166–4.578, *p* = 0.016) (23) | Active dissemination of online health information appeared to be associated with higher digital health literacy among stroke patients. |
|  |  | Willingness to interact with individuals with mental illness | Quantitative | Compared with the low e-health literacy group, participants who were unwilling to interact with people with mental illness (OR = 2.019, *p* = 0.006) were more likely to enter the low application-high decision-making group (21) | Willingness to interact with individuals with mental illness appeared to be associated with digital health literacy profiles among stroke patients. |
